# The role of ultrasound of the quadriceps femoris in people with cystic fibrosis: a systematic review and meta-analysis

**DOI:** 10.36416/1806-3756/e20250232

**Published:** 2026-03-05

**Authors:** Jorge Eduardo Cortz Sernaglia, Aline Priscila de Souza, Aline Cristina Gonçalves, Daniela de Souza Paiva Borgli, Carla Cristina Souza Gomez, José Dirceu Ribeiro

**Affiliations:** 1. Faculdade de Ciências Médicas, Universidade Estadual de Campinas - UNICAMP - Campinas (SP) Brasil.

**Keywords:** Ultrasonography, Cystic fibrosis, Quadriceps muscle

## Abstract

**Objective:**

To conduct a systematic review and meta-analysis of the evidence on the use of ultrasound to assess quadriceps morphology in people with cystic fibrosis (PwCF), focusing on muscle thickness, cross-sectional area, and associations with clinical outcomes.

**Methods:**

This study followed the Preferred Reporting Items for Systematic Reviews and Meta-Analyses guidelines and was registered on the International Prospective Register of Systematic Reviews platform (ID: CRD42024590549). The PubMed, Embase, Scopus, Web of Science, SciELO, and Cumulative Index to Nursing and Allied Health Literature databases were comprehensively searched without time restrictions. Gray literature and manual reference screening were also included. Observational studies assessing quadriceps morphology via ultrasound in people with CF and reporting quantitative outcomes were eligible. Two reviewers independently conducted study selection, data extraction, and risk of bias assessment using the Joanna Briggs Institute tool. Meta-analysis was performed with Cochrane’s Review Manager software.

**Results:**

Five studies met the inclusion criteria. People with CF showed reduced quadriceps thickness and cross-sectional area, particularly those who were malnourished. Moderate correlations were observed between ultrasound measurements and clinical parameters such as pulmonary function, fat-free mass index, and muscle strength. Meta-analysis revealed a significant reduction in rectus femoris muscle thickness in people with CF when compared with controls (mean difference: −0.50 cm; 95% CI, −0.78 to −0.22; p = 0.0004), with high heterogeneity (I^2^ = 94%). Risk of bias was moderate because of small sample sizes and methodological variability.

**Conclusions:**

Although the evidence is limited and heterogeneous, people with CF appear to show reduced quadriceps morphology, with associations with nutritional and functional status. Further high-quality studies are needed to confirm our findings and determine the clinical utility of ultrasound in this population.

## INTRODUCTION

Cystic fibrosis (CF) is an autosomal recessive genetic disease caused by mutations in the CF transmembrane conductance regulator (*CFTR*) gene, resulting in dysfunction of a transmembrane protein that is essential for chloride and bicarbonate transport. This impairment disrupts epithelial homeostasis in multiple organs.^1^ As a result, people with CF produce thick, sticky mucus that is poorly cleared from the lungs, promoting persistent retention and, consequently, chronic and progressive lung damage.^2^

Skeletal muscle deficit is a significant clinical feature of chronic respiratory diseases and a persistent hallmark of CF.^3^ Muscle weakness in patients with CF is closely linked to severe complications, including reduced aerobic capacity, which impairs quality of life and is one of the strongest predictors of prognosis and survival.^4^ The quadriceps, a key muscle for functional capacity, is strongly associated with maximal aerobic performance. Its weakness, combined with fat-free mass loss, compromises cardiopulmonary exercise test performance, contributing to a progressive decline in quality of life and increased mortality.^5^ ^-^ ^8^

Muscle mass reduction in people with CF is multifactorial, driven by physical inactivity, systemic inflammation, CFTR dysfunction in skeletal muscle, hormonal imbalances (e.g., low IGF-I levels), and corticosteroid side effects. Additionally, frequent exacerbations, oxidative stress, and malnutrition contribute to muscle atrophy, predominantly affecting the lower limbs.^4^ People with CF also experience significant quadriceps weakness, a hallmark of peripheral muscle impairment in such patients. This decline in strength is not solely attributed to physical inactivity but also to systemic inflammation and metabolic disturbances.^9^ ^,^ ^10^

Ultrasound has been widely studied as a promising tool for skeletal muscle monitoring, particularly in vulnerable populations, because of its accessibility and absence of radiation. Research has compared ultrasound with imaging techniques that are more complex, such as CT.^11^ As a noninvasive, portable, and cost-effective method, ultrasound is a viable alternative to modalities such as dual-energy X-ray absorptiometry (DEXA).^12^ Among the muscles assessed via ultrasound, the quadriceps femoris is commonly studied because of its functional relevance and ease of visualization. Its bipennate structure, featuring a distinct central intramuscular tendon, enables precise muscle thickness measurements.^13^ Additionally, the rectus femoris, given its superficial location, can be easily identified by using ultrasound, thus facilitating longitudinal monitoring of muscle changes.^14^

Ultrasound has been explored as a potential tool for assessing the quadriceps in people with CF. However, its clinical applicability in detecting muscle alterations has yet to be fully established. Therefore, understanding its feasibility and efficacy in this context may contribute to improving muscle mass monitoring strategies in people with CF.^15^ ^-^ ^17^

The objective of the present systematic review and meta-analysis was to assess the diagnostic efficacy and feasibility of ultrasound of the quadriceps femoris in people with CF, the advantages and limitations of the method being investigated either alone or in comparison with traditional imaging methods such as DEXA and CT. 

## METHODS

We searched the Cumulative Index to Nursing and Allied Health Literature, Embase, PubMed, SciELO, Scopus, and Web of Science databases using terms such as “cystic fibrosis,” “ultrasound,” “quadriceps muscle,” and variations thereof based on DeCS. No time restrictions were used. Gray literature, including records from ClinicalTrials.gov, was also searched. Additionally, a manual search was performed within the reference lists of the included studies. Table S1 (supplementary material) presents the search strategies used in all databases. The last search was conducted on April 13, 2025. We formulated the following Patients of interest, Intervention to be studied, Comparison of intervention, and Outcome of interest question: In individuals with CF (P), what is the efficacy and feasibility of ultrasound (I), either alone or in comparison with other imaging exams (C), in detecting changes in the quadriceps femoris (O)? The present systematic review and meta-analysis was registered on the International Prospective Register of Systematic Reviews platform (ID: CRD42024590549). Table S2 (supplementary material) shows the Preferred Reporting Items for Systematic Reviews and Meta-Analyses checklist. 

### Eligibility criteria

The inclusion criteria for the present study were as follows: empirical observational studies, including cohort studies, cross-sectional studies, and case reports when applicable; studies involving people with CF in various age groups (including children, adolescents, and adults); studies performing ultrasound evaluation of the quadriceps femoris, either alone or in comparison with other imaging or body composition assessment methods such as bioelectrical impedance analysis, DEXA, CT, and magnetic resonance imaging; studies reporting quantitative outcomes of interest, including muscle thickness (reported in millimeters or centimeters), cross-sectional area (in cm^2^), muscle mass (in kilograms), and muscle quality as assessed by echogenicity in grayscale units; studies providing original data related to quadriceps morphology or function as assessed by ultrasound; and studies published in English or Portuguese. 

Exclusion criteria were as follows: systematic reviews, meta-analyses, narrative reviews, editorials, letters, and theoretical or conceptual papers; studies that did not focus on quadriceps femoris evaluation; studies that did not use ultrasound as an assessment method; and ongoing studies. 

### Selection process

The Rayyan tool was used to select the articles found in each database. The selection process was conducted in a blind, paired manner, in accordance with the inclusion criteria. Any discrepancies regarding the inclusion or exclusion of articles were discussed with a third author. 

### Data collection process

Data were collected with a structured Excel spreadsheet, including information such as article title and year of publication; names of the authors and research groups; sample characteristics (including age and sex); methods (e.g., ultrasound and DEXA); relevant statistics; main findings; limitations; clinical implications; future trends; discussion of the results; conclusions; biases; and conflicts of interest. Two reviewers independently extracted the data and then compared their entries. In case of discrepancies, the reviewers discussed until consensus was reached, thus ensuring data accuracy and reliability without needing a third reviewer. 

### Data items

Data on quadriceps thickness were analyzed in order to assess the condition of the peripheral muscles in people with CF. Possible correlations between quadriceps thickness and outcomes such as muscle strength, body composition, and disease severity were also examined. Study variables included rectus femoris muscle area, fat-free mass index as assessed by DEXA, bioelectrical impedance analysis, and anthropometry, as well as handgrip strength and pulmonary function. Muscle contraction and echogenicity were also analyzed as muscle quality and function indicators. 

Additional information regarding participant characteristics (e.g., age, sex, and clinical status), details of the interventions, and study funding sources was considered when available. 

### Study risk of bias assessment

Two independent reviewers conducted the risk of bias assessment using the Joanna Briggs Institute tool, with the appropriate version applied to each type of included study. To enhance clarity and facilitate interpretation, the results were summarized by using the traffic light plot format generated by the robvis tool, which provides a visual synthesis of the risk of bias across studies. 

### Effect measures

For outcome analysis, the Review Manager software, version 5.4 (RevMan 5; Cochrane Collaboration, Oxford, United Kingdom) was used in order to calculate effect measures for the control and intervention groups. The analyses included mean difference, 95% confidence interval, fixed effects, and inverse variance, thus ensuring a structured and reliable evaluation of the results. The studies were categorized on the basis of their comparison groups, allowing an appropriate synthesis that minimized potential biases. 

### Synthesis methods

Studies were included if they reported on the efficacy and feasibility of ultrasound in assessing the quadriceps femoris in people with CF. Eligibility was determined by evaluating the study design, the study population, and the reported outcomes. Studies were then grouped on the basis of the similarity of their methods and objectives, thus ensuring consistency in the synthesis process. 

When studies did not provide means and standard deviations but instead reported medians and interquartile ranges, as well as minimum and maximum values, a qualitative analysis was performed. This decision was made because RevMan 5.4 requires means and standard deviations for statistical calculations. Data conversions were considered; however, when not feasible, the studies were excluded from the quantitative synthesis and analyzed qualitatively. 

All extracted data were tabulated in Excel, with the key findings being consolidated into a single table. To synthesize the results, studies were grouped on the basis of their primary outcome measures and analyzed by using forest plots in RevMan 5.4. To address heterogeneity, subgroup analyses were conducted whenever sufficient data were available. This allowed a comparison of studies with similar designs, intervention protocols, and outcome measures. In cases in which heterogeneity remained high or in which subgrouping was not feasible, a qualitative approach was used in order to interpret the findings. Therefore, traditional sensitivity analyses were not conducted. 

### Reporting bias assessment

The Grading of Recommendations Assessment, Development, and Evaluation (GRADE) approach was used in order to assess the quality of evidence and to support the development of summary of findings tables, with the aid of the GRADEpro GDT online tool (available from https://www.gradepro.org/). 

## RESULTS

**Figure 1:**
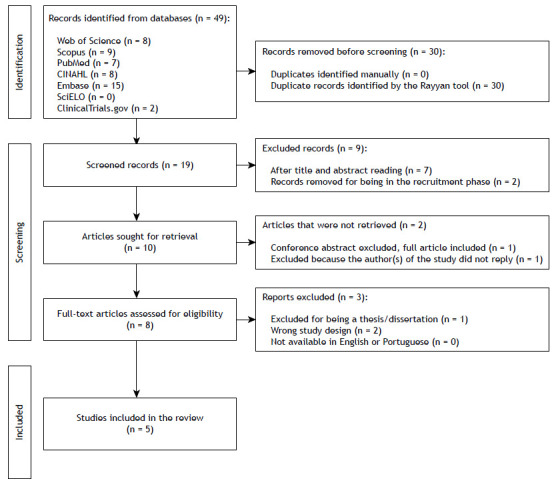
Preferred Reporting Items for Systematic Reviews and Meta-Analyses flow diagram for study selection. CINAHL: Cumulative Index to Nursing and Allied Health Literature.

A total of 49 studies were retrieved from the databases. Of those 49 studies, 30 were removed before screening for being duplicates. Of the remaining 19, 9 were excluded after title and abstract screening. A total of 10 studies were selected for full-text reading, but 2 could not be retrieved: 1 because it was a conference abstract (the original article having been included) and 1 because the authors did not reply to our request. Of the 8 studies assessed for eligibility, 3 were excluded because of inappropriate study design: 1 was excluded because it was a thesis/dissertation, and 2 were excluded because of inappropriate study design. Therefore, 5 studies were included in the review.^18^ ^-^ ^22^ Figure 1 presents the Preferred Reporting Items for Systematic Reviews and Meta-Analyses flow diagram for study selection. 

Four of the 5 studies included in the present review involved adult CF patients.^18^ ^,^ ^20^ ^-^ ^22^ One study was conducted in Brazil,^19^ 1 was conducted in Spain,^18^ 1 was conducted in Turkey,^22^ and 2 were conducted in Canada.^20^ ^,^ ^21^ Table 1 presents a summary of the 5 included studies. None of the studies reported the reference equations used for assessing quadriceps thickness or cross-sectional area values. 

**Table 1:** Synthesis of the findings of the studies included in the present systematic review and meta-analysis.

Authors	Objectives	Population	Methods	Outcomes	Ultrasound measurements and associations	Main results	Conclusions
Souza et al.^19^ Brazil	To compare muscle thickness and subcutaneous fat between pediatric CF patients and healthy controls	84 children and adolescents (39 with CF and 45 healthy controls; age: 6-18 years)	Controlled cross-sectional study with ultrasound for quadriceps assessment and spirometry for pulmonary function in CF patients and controls	Muscle thickness and subcutaneous fat in different muscles: triceps brachii, quadriceps femoris, and medial gastrocnemius	Absolute values	Patients with CF had significantly lower BMI, calf circumference, and femur diameter; moderate correlation between quadriceps thickness and FVC.	Quadriceps ultrasound was useful in correlating pulmonary function and nutritional parameters in young people with CF.
Sánchez-Torralvo et al.^18^ Spain	To assess the effectiveness of quadriceps ultrasound as a nutritional assessment tool in adults with CF	48 adults with confirmed CF	Prospective observational study with quadriceps ultrasound being compared with BIA, DEXA, and handgrip strength	Nutritional status, lean mass, and muscle strength	Absolute values	Strong correlation between quadriceps ultrasound measurements and other body composition measures such as BIA and DEXA; positive relationship with nutritional status	Quadriceps ultrasound proved to be a reliable and practical tool for nutritional assessment in adults with CF, with a high correlation with standard methods such as BMI, skinfold thickness, mid-upper arm circumference, adductor pollicis muscle area, and BIA
Wu et al.^21^ Canada	To assess the role of CFTR in skeletal muscle contractility in patients with CF	52 adults with CF (34 minimal mutations and 18 residual mutations)	Cross-sectional study. Muscle strength, power, and size were measured by dynamometry, stair climb, and ultrasound, respectively. Regression analysis was used in order to adjust for confounders.	Although torque was similar between groups, contractility (torque per thickness/area) was lower in the minimal function group.	Force normalized by thickness/area; absolute values	CFTR function influences muscle contractility. Minimal function mutations lead to reduced force relative to muscle size.	Quadriceps thickness was preserved, but contractility was reduced in minimal function CFTR, indicating impaired muscle quality despite normal size.
Wu et al.^20^ Canada	To compare the quadriceps size and quality in adults with CF with different levels of CFTR dysfunction	52 adults (34 with severe CFTR dysfunction and 18 with mild CFTR dysfunction)	Prospective, cross-sectional study with ultrasound for RF-CSA and RF-ECHO	Quadriceps size (RF-CSA) and quality (RF-ECHO)	Force adjusted by thickness/area; absolute values	Individuals with severe dysfunction had larger RF-CSA by 3.22 cm^2^. No differences in quadriceps thickness or echogenicity (RF-ECHO) were observed between groups.	Ultrasound was useful for distinguishing quadriceps size across different levels of CFTR dysfunction, although with no significant impact on muscle quality.
Uslu et al.^22^ Turkey	To evaluate muscle thickness, handgrip strength, and diaphragm function in adults with CF	54 adults (31 with CF and 23 healthy controls; age: 18-45 years)	Cross-sectional study with ultrasound for quadriceps and diaphragm; handgrip strength test; and the six-minute walk test	Quadriceps thickness, handgrip strength, and diaphragm function	Absolute values	People with CF had thinner quadriceps and lower handgrip strength than did healthy controls.	Quadriceps ultrasound showed muscle thinning in people with CF, demonstrating its usefulness for monitoring peripheral muscle loss and functionality.

CF: cystic fibrosis; BIA: bioelectrical impedance analysis; DEXA: dual-energy X-ray absorptiometry; CFTR: cystic fibrosis transmembrane conductance regulator; RF-CSA: rectus femoris cross-sectional area; and RF-ECHO: rectus femoris echogenicity.

### Muscle changes as detected by ultrasound

Three studies(18,19,22) reported changes in quadriceps circumference and rectus femoris muscle thickness as measured by ultrasound in people with CF, including reductions in muscle size or thickness. Additionally, Souza et al.(18) reported that quadriceps thickness was positively correlated with FVC (r = 0.336; p = 0.05) and lean body mass (r = 0.424; p = 0.01), despite not finding significant between-group differences in muscle thickness. 

**Figure 2:**
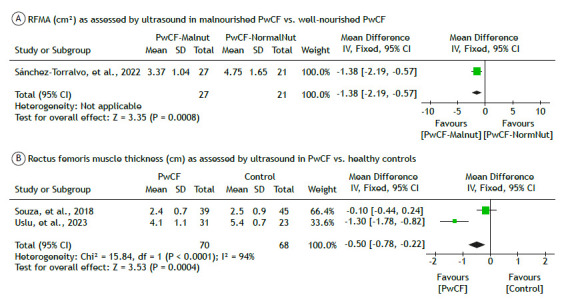
Forest plot of rectus femoris muscle area (RFMA) and rectus femoris muscle thickness as assessed by ultrasound. In A, comparison between malnourished people with cystic fibrosis (PwCF) and their nutritionally normal counterparts (controls) revealing a significant reduction in rectus femoris muscle thickness and RFMA in the malnourished group (mean difference: −1.38 cm; 95% CI, -2.19 to −0.57; p = 0.0008). In B, comparison between PwCF (independently of nutritional status) and healthy controls revealing a significant reduction in rectus femoris muscle thickness in PwCF (mean difference: −0.50 cm; 95% CI, −0.78 to −0.22; p = 0.0004), with substantial heterogeneity being observed (I^2^ = 94%). These findings highlight the negative impact of cystic fibrosis and malnutrition on quadriceps muscle morphology as quantified by ultrasound.

Figure 2 shows a forest plot illustrating the mean difference in rectus femoris muscle area (in cm^2^) and rectus femoris muscle thickness (in cm) as assessed by ultrasound across two comparative analyses. 

Wu et al.(21) reported that participants with residual function *CFTR* mutations showed significantly greater quadriceps muscle contractility than did those with minimal function mutations. Quadriceps torque normalized to quadriceps layer thickness was 27.5 Nm/cm higher (95% CI, 2.2-52.8 Nm/cm; p = 0.034), and torque normalized to rectus femoris cross-sectional area was 5.6 Nm/cm² higher (95% CI, 0.3-10.9 Nm/cm^2^; p = 0.041). 

### Correlations of muscle ultrasound metrics with body composition, strength, and pulmonary function

Sánchez-Torralvo et al.(18) reported a strong correlation between rectus femoris muscle area and fat-free mass as assessed by anthropometry (r = 0.747; p = 0.001), DEXA (r = 0.678; p = 0.001), fat-free mass index (r = 0.712; p = 0.001), bioelectrical impedance analysis (r = 0.780; p = 0.001), and handgrip strength (r = 0.790; p = 0.001), as well as a moderate correlation with pulmonary function as assessed by percent predicted FEV_1_ (FEV_1_%; r = 0.445; p = 0.005). In this context, a significantly lower rectus femoris muscle area was observed in malnourished people with CF when compared with individuals with normal nutritional status (4.75 ± 1.65 cm^2^ vs. 3.37 ± 1.04 cm^2^; p = 0.014), according to Sánchez-Torralvo et al.(18). 

Uslu et al.(22) found lower thickness of the right rectus femoris muscle in people with CF when compared with controls (4.1 ± 1.1 vs. 5.4 ± 0.7; p = 0.001), with a correlation with BMI (r = 0.569; p = 0.001). They also reported that handgrip strength was significantly correlated with fat-free mass index (r = 0.497; p = 0.004) and pulmonary function test values (FEV_1_: r = 0.443, p =0.013; FVC: r = 0.582, p = 0.001; and percent predicted FVC: r = 0.377, p = 0.037). 

Wu et al.(20) reported a correlation between rectus femoris cross-sectional area and FEV_1_% (r = 0.46; p = 0.01), as well as between rectus femoris cross-sectional area and quadriceps strength (r = 0.43; p = 0.02). The mean rectus femoris cross-sectional area was 10.6 ± 3.1 cm^2^ in the residual *CFTR* group and 13.8 ± 3.5 cm^2^ in the minimal function group. No significant correlation was found between muscle echogenicity and FEV_1_% (r = −0.16; p = 0.36) or quadriceps strength. 

### Clinical feasibility of ultrasound in people with cystic fibrosis

Sánchez-Torralvo et al.(18) described ultrasound as a simple, radiation-free tool that can be routinely applied in clinical practice. Souza et al.(19) stated that ultrasound is a low-cost, noninvasive method, feasible even in people with limited mobility. Wu et al.(21) explicitly reinforced these characteristics, referring to ultrasound as a noninvasive, low-cost, and low-risk technique suitable for broader application in studies involving people with CF. 

Table 2 summarizes the information reported in the included studies regarding ultrasound equipment and image acquisition protocols. None of the studies provided information on the time required for examination or interobserver evaluation. 

**Table 2:** Ultrasound equipment and image acquisition protocols used across studies.

Study	Device	Probe Frequency	Gain	Depth	Patient Positioning	Image Mode and Analysis	Muscles studied	Anatomical reference point for measurement	Number of images for averaging and discarding	Minimum variation
Souza et al.^19^	DP-6600 (Mindray)	7.5 MHz	Not reported	Not reported	Limbs relaxed and extended	B-mode, ImageJ software	quadriceps	Midpoint between the ASIS and the patella.	Not reported	Same evaluator, trained by an experienced investigator
Sánchez-Torralvo et al.^18^	GE Logiq P5	7-13 MHz linear probe	55-60 dB	4-6 cm	Supine position, relaxed lower limbs	B-mode, images analyzed using ImageJ software	rectus femoris	Trochanter-patella distance and rectus femoris thickness at midpoint	3 images per rectus femoris measurement point; number of discarded images not reported	Same evaluator, familiar with the technique
Uslu et al.^22^	GE Healthcare	10-13 MHz, standardized at 12 MHz	32 dB (fixed)	5.5 cm	Midpoint between greater trochanter and patella	B-mode, not specified	rectus femoris	Midpoint between the ASIS and the patella.	3 measurements of the rectus femoris thickness for each side of the quadriceps; number of discarded images not reported	Not reported
Wu et al.^20^	GE Logiq E	8-12 MHz (CSA), up to 13 MHz (thickness)	56-98 dB	4.5-9 cm	Supine, knee flexed at 30°, supported by pillow	B-mode, not specified	rectus femoris, vastus lateralis, and vastus intermedius	Midpoint between the ASIS and the patella.	3 images for each region of the quadriceps; number of discarded images not reported	Mean inter-rater difference: 0.47 cm^2^ for RF-CSA; thickness difference: 0.004-0.05 cm; within 95% limits of agreement
Wu et al.^21^	GE Logiq E	8-12 MHz (CSA), up to 13 MHz (thickness)	56-98 dB	4.5-9 cm	Supine, knee flexed at 30°, supported by pillow	B-mode, not specified	rectus femoris, vastus lateralis, and vastus intermedius	Midpoint between the ASIS and the patella.	3 images for measurements of quadriceps and biceps brachii; number of discarded images not reported	Not reported

CSA: cross-sectional area; B-mode: brightness mode; ImageJ: Image analysis software (National Institutes of Health, Bethesda, MD, USA); RF-CSA: rectus femoris cross-sectional area; and ASIS: anterior superior iliac spine.

### Quality of evidence and limitations of the studies

Of the 5 studies analyzed, 4 explicitly declared no conflicts of interest.(19-22) Only Sánchez-Torralvo et al.(18) did not provide a conflict of interest statement. Uslu et al.(22), Sánchez-Torralvo et al.(18), and Souza et al.(19) did not address potential sources of bias or reflect on methodological limitations, which could impact the interpretation and generalizability of their findings. Wu et al.(20) and Wu et al.(21) addressed potential sources of bias, although neither group of authors applied a standardized assessment tool. Their evaluations were narrative, focusing on limitations such as small sample size and possible confounding factors. None of the studies used structured instruments for systematic bias assessment. 

Sánchez-Torralvo et al.(18) did not examine correlations between quadriceps thickness and disease severity. Wu et al.(20) did not evaluate the clinical utility, diagnostic accuracy, or feasibility of ultrasound for assessing muscle structure in this context. 

Regarding cost-related and logistical considerations, none of the 5 studies conducted a comparative assessment of the cost-effectiveness of ultrasound, nor did they report the time required to perform the examination. 

Table S3 (supplementary material) presents the results of the certainty of evidence analysis with the GRADEpro GDT online tool (available from https://gdt.gradepro.org/app/). 

A comprehensive selection of relevant studies on the topic was included to represent the available evidence. Because fewer than ten studies were identified, the use of statistical methods to assess risk of bias due to missing results, such as funnel plots, is limited. However, the studies analyzed provide a consistent basis for the findings. It is important to note that, despite these methodological limitations, the conclusions reflect the current understanding based on the available data. 

**Figure 3:**
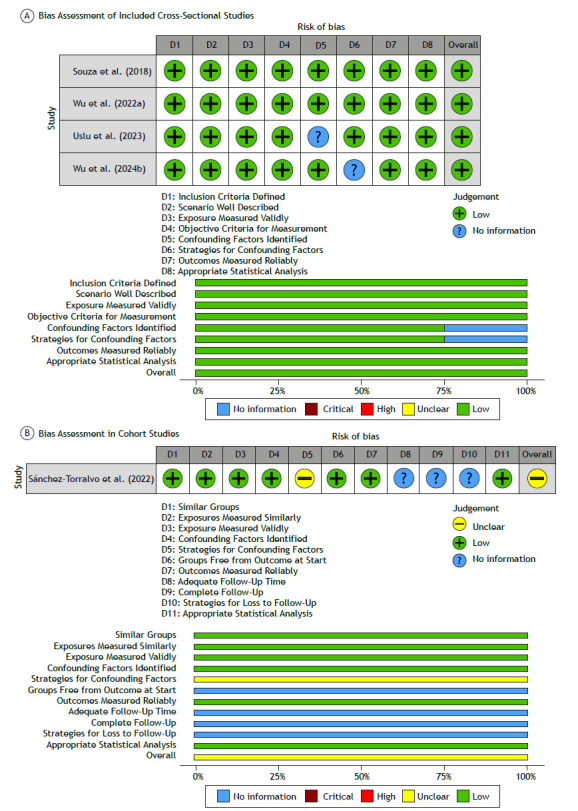
Bias assessment with traffic light plot and summary (the Joanna Briggs Institute tool).

Figure 3 shows the assessment of risk of bias using a generic traffic light graph adapted from the Joanna Briggs Institute tool. 

## DISCUSSION

This systematic review and meta-analysis highlighted the relevance of ultrasound in the assessment of the quadriceps femoris in people with CF, addressing morphological aspects (muscle thickness and cross-sectional area) and qualitative aspects (echogenicity), as well as their correlations with nutritional, functional, and respiratory variables. 

Significant reductions in rectus femoris muscle thickness were observed in malnourished people with CF when compared with controls with normal nutritional status (mean difference: −1.38 cm; 95% CI, −2.19 to −0.57; p = 0.0008), supporting the sensitivity of ultrasound in detecting muscle loss associated with nutritional status (Figure 2). This finding is particularly relevant in the context of chronic pulmonary diseases, in which muscle wasting is common and directly impacts functional capacity and clinical outcomes. 

In addition to the findings in people with CF, the literature on other chronic diseases provides important evidence that reinforces the clinical potential of quadriceps ultrasound as a tool for morphofunctional and prognostic assessment. In people with CF, this potential is supported by strong correlations between muscle ultrasound metrics and functional parameters, such as handgrip strength (r = 0.790; p = 0.001) and pulmonary function (FEV_1_%: r = 0.445; p = 0.005).(18)

In patients with COPD, the quadriceps muscle contractile index, as assessed by ultrasound, showed significant associations with clinically relevant outcomes. Positive correlations were observed between the quadriceps muscle contractile index and fat-free mass index (ρ = 0.59; p = 0.001), FEV_1_% (ρ = 0.43; p = 0.001), and diaphragmatic contractility (ρ = 0.41; p = 0.008), whereas a negative correlation was found with symptom burden as measured by the COPD Assessment Test score (ρ = −0.47; p = 0.002).^23^ These findings support the role of quadriceps ultrasound as a sensitive and noninvasive tool for assessing muscle status and disease severity in patients with chronic diseases.^23^ ^-^ ^25^

Quadriceps strength was found to be significantly reduced in COPD patients when compared with controls (p < 0.001), correlating positively with FVC (r^2^ = 0.33; p < 0.005) and FEV_1_% (r^2^ = 0.37; p < 0.005) and highlighting the link between lung function and muscle strength.^26^

Similarly, correlations between thickness of the right rectus femoris and BMI (r = 0.569; p = 0.001), as well as between muscle cross-sectional area and quadriceps strength, have been observed in people with CF, suggesting a parallel role of ultrasound in assessing muscle function in patients with chronic respiratory diseases.^20^ ^,^ ^22^

Studies have shown high intra- and interobserver reliability, with intraclass correlation coefficient values ranging from 0.72 to 1.000.^27^ Furthermore, its portability and bedside applicability, even in frail populations, are noteworthy advantages.^27^ Specifically, ultrasound of the quadriceps, particularly the vastus lateralis muscle, has been shown to be a reliable and valid technique for muscle assessment, showing excellent reproducibility (with an intraclass correlation coefficient ranging from 0.929 to 0.994; p < 0.0001) and good agreement with magnetic resonance imaging (r^2^ = 0.793; p < 0.0001).^28^

The included studies also highlight ultrasound as a simple, radiation-free, low-risk technique that is feasible even in people with limited mobility, ultrasound being easy to use in routine CF clinical practice.^18^ ^,^ ^19^ ^,^ ^21^

Several studies including different populations with chronic diseases reinforce the clinical relevance of muscle ultrasound, demonstrating significant correlations between muscle thickness and clinical and functional parameters such as strength, pulmonary function, physical performance, and body composition. These findings provide important support for the results of the present review, especially in the context of CF. 

The findings of the present review, including a positive correlation of rectus femoris cross-sectional area with FEV_1_% (r = 0.46; p = 0.01), further corroborate the growing evidence that ultrasound reflects muscle health and function in people with CF.^20^

The echo intensity of the rectus femoris was negatively associated with the number of daily steps (β = −0.657; p = 0.001) and moderate-to-vigorous physical activity time (β = −0.590; p = 0.001), whereas muscle cross-sectional area was positively associated with knee extensor strength (β = 0.553; p = 0.004).^29^ These findings align with previously reported inverse relationships between muscle quality (echogenicity) and physical activity levels, as well as with a positive correlation between muscle size and strength in other studies involving people with CF.^18^

Greater rectus femoris thickness and cross-sectional area showed significant positive correlations with fat-free mass index (r = 0.53-0.57; p ≤ 0.003) and handgrip strength (r = 0.48-0.58; p ≤ 0.009). Handgrip strength showed a positive and significant correlation with muscle thickness (r = 0.48; p = 0.009) and muscle cross-sectional area (r = 0.58; p < 0.001).^30^ These findings reinforce the strong association between muscle morphology and functional strength in people with CF, supporting evidence presented by Uslu et al.^22^ and Wu et al.^20^ In the study by Wu et al.,^20^ individuals with more severe CFTR dysfunction exhibited a larger rectus femoris cross-sectional area, a finding that contrasts with intuitive expectations. According to our risk of bias assessment, that study was rated as low risk; however, the overall certainty of evidence (GRADE) for this outcome was low. A biologically plausible explanation is that adults with more severe CF may present greater intramuscular fat infiltration, which can increase measured cross-sectional area without reflecting preservation or hypertrophy of contractile tissue.^31^ This pattern has been described in chronic disease populations and may be influenced by nutritional status.^32^

Quadriceps muscle strength (65.8%) correlated with walk distance (r = 0.54), peak oxygen consumption (volume of oxygen; r = 0.72), lean body mass (r = 0.83), and BMI (r = 0.69); handgrip strength correlated with BMI (r = 0.45) and lean body mass (r = 0.65). Functional capacity (percent predicted walk distance) was higher in stronger muscle groups (p = 0.02), highlighting its impact on people with CF.^31^ CF represents a clinical scenario with a high risk of malnutrition, in which the preservation of muscle mass is an important prognostic marker.^33^ ^-^ ^35^ This is supported by a significantly lower rectus femoris muscle area in malnourished people with CF when compared with individuals with normal nutritional status (4.75 ± 1.65 cm^2^ vs. 3.37 ± 1.04 cm^2^; p = 0.014), as well as by reductions in quadriceps thickness in comparison with controls (4.1 ± 1.1 cm vs. 5.4 ± 0.7 cm; p = 0.001).^18^ ^,^ ^20^ Although studies investigating people with CF remain limited, evidence from other populations reinforces the ability of this technique to detect skeletal muscle alterations associated with malnutrition. 

In critically ill people with moderate malnutrition, a significant reduction in quadriceps thickness was observed (1.46 ± 0.46 cm), with significant differences between nutritional status groups (p = 0.023).^36^ These results reinforce that muscle ultrasound offers a quantitative, sensitive, and clinically applicable approach across different levels of care, from initial diagnosis to follow-up of patients at high nutritional risk, as is the case with CF patients. 

The effects of CFTR modulators on body composition have been the focus of growing interest in the literature, with evidence showing that the benefits go beyond simple weight gain. Wu et al.^21^ demonstrated that after one year of treatment with elexacaftor/tezacaftor/ivacaftor, people with CF experienced a significant increase in both total and appendicular lean mass, with an average gain of 1.8 kg of lean mass. Additionally, improvements in pulmonary function and inflammatory markers reinforced the hypothesis of a positive systemic effect of these modulators on muscle metabolism.^37^ ^-^ ^40^ One study using CT analyzed body composition in adults with CF and reported a modest but significant increase in muscle mass (of 1.63%; p = 0.008) after initiation of elexacaftor/tezacaftor/ivacaftor therapy.^41^ In contrast, there was a much more pronounced gain in body fat reserves, especially among previously underweight patients. The increase in muscle mass was correlated with improved pulmonary function (r = 0.360; p = 0.004), highlighting the multifactorial effects of modulator therapy.^41^ These findings align with observed higher quadriceps torque normalized to muscle thickness and rectus femoris cross-sectional area in participants with residual function *CFTR* mutations (27.5 Nm/cm, p = 0.034; 5.6 Nm/cm^2^, p = 0.041).^21^ In addition, data from a cohort of 234 children and adolescents (mean age, 13.6 years) showed that after 6 and 12 months of CFTR modulator therapy, there were significant increases in weight and BMI Z-scores, as well as increases in fat mass, both in absolute values (kg) and percentage (p < 0.05). Fat-free mass also increased significantly at both time points (p < 0.05).^42^ These results indicate that CFTR modulators promote significant gains in body composition in the pediatric population, including fat and lean mass gains. 

With the changing clinical profile of people with CF, driven by the introduction of CFTR modulators, new care challenges are emerging that require more than the simple maintenance of respiratory function. The literature highlights that ultrasound of the quadriceps femoris allows assessment of muscle mass, quality, and architecture,^43^ ^-^ ^46^ contributing to an increased understanding of the functional status of people with CF. Amidst these changes, it may be time to reflect on the future role of professionals traditionally associated solely with ventilatory management, in the face of an increasingly prominent demand for morphofunctional assessment and broader clinical reasoning. 

The results showed high statistical heterogeneity, with I^2^ values reaching up to 94% in some analyses. Possible causes include differences between the studied populations, such as age, disease severity, and *CFTR* genotypes, variations in ultrasound protocols (e.g., equipment, probe frequency, patient positioning, and muscles evaluated), and small sample sizes. Because ultrasound examinations depend on the operator, the need for standardized training and acquisition procedures is evident. Differences in acquisition protocols and in the choice of muscle or anatomical region can substantially influence the results and hinder comparisons between studies; therefore, we performed two separate statistical analyses for different muscle groups (Figure 2). In addition, the absence of healthy control groups with matching age and sex in some studies, together with the wide age range observed, imposes methodological limitations, particularly when using absolute values for comparisons. This heterogeneity reduces confidence in pooled estimates, highlighting the urgent need for methodological standardization of the technical aspects of ultrasound and statistical criteria. The adoption of uniform protocols can help reduce heterogeneity and strengthen the quality of evidence. 

A relevant limitation of the studies included in the present systematic review and meta-analysis was the absence of formal risk of bias assessment, with none of the studies having employed tools such as the Newcastle-Ottawa Scale or Risk Of Bias In Non-randomized Studies - of Interventions. Most of the studies had small sample sizes, lacked control for confounding variables, and did not implement blinding procedures, thus increasing the risk of selection and measurement bias. There was no prior protocol registration or detailed description of strategies to minimize bias. To address these limitations, this review incorporated the use of the GRADE system as an additional strategy to assess more systematically the overall quality of the evidence. 

We acknowledge the scarcity of studies specifically involving people with CF, which limits the direct extrapolation of results to this population. Additionally, methodological heterogeneity made it difficult to compare findings across studies. We also observed a lack of more standardized study designs, such as multicenter validation studies. Another concern is the potential risk of publication bias, given that most studies reported positive results. To the best of our knowledge, this is the first review compiling the main ultrasound modes and techniques used to assess muscle mass in people with CF. This study is useful for guiding future research by identifying protocols and parameters applicable to other clinical contexts. 

Ultrasound is practical, safe, and free from radiation, allowing repeated assessments without risk. It consistently correlates with nutritional and functional parameters, such as muscle strength. Therefore, it could serve as a valuable tool in clinical evaluation.^47^ ^,^ ^48^

None of the studies addressed aspects related to time, cost-effectiveness, or reliability of ultrasound assessment. Although current evidence specific to CF is still limited, findings from other chronic diseases underscore the promising potential of quadriceps ultrasound as a sensitive and clinically relevant assessment tool. Its noninvasive nature and ability to detect muscle alterations make it a valuable technique, warranting further investigation in people with CF. Given that none of the studies reviewed assessed cost-effectiveness, this aspect remains to be explored in future research. Well-designed and robust studies are urgently needed to clarify the clinical utility of ultrasound of the quadriceps femoris in people with CF. 
